# LACTB, a Metabolic Therapeutic Target in Clinical Cancer Application

**DOI:** 10.3390/cells11172749

**Published:** 2022-09-03

**Authors:** Xiaohua Li, Zhongkai Ren, Xiaohong Huang, Tengbo Yu

**Affiliations:** 1 School of Basic Medical Sciences, Qingdao University, Qingdao 266071, China or; 2 Department of Sports Medicine, The Affiliated Hospital of Qingdao University, Qingdao 266000, China or; 3 Shandong Institute of Traumatic Orthopedics, Medical Research Center, The Affiliated Hospital of Qingdao University, Qingdao 266590, China; 4 Institute of Sports Medicine and Rehabilitation, Qingdao University, Qingdao 266071, China

**Keywords:** serine beta-lactamase-like protein, mitochondrial intermembrane, lipid metabolism, tumor suppressor, therapeutic target, prognosis

## Abstract

Serine beta-lactamase-like protein (LACTB) is the only mammalian mitochondrial homolog evolved from penicillin-binding proteins and β-lactamases (PBP-βLs) in bacteria. LACTB, an active-site serine protease, polymerizes into stable filaments, which are localized to the intermembrane space (IMS) of mitochondrion and involved in the submitochondrial organization, modulating mitochondrial lipid metabolism. Cancer pathogenesis and progression are relevant to the alterations in mitochondrial metabolism. Metabolic reprogramming contributes to cancer cell behavior. This article (1) evidences the clinical implications of LACTB on neoplastic cell proliferation and migration and tumor growth and metastasis as well as LACTB’s involvement in chemotherapeutic and immunotherapeutic responses; (2) sketches the structural basis for LACTB activity and function; and (3) highlights the relevant regulatory mechanisms to LACTB. The abnormal expression of LACTB has been associated with clinicopathological features of cancer tissues and outcomes of anticancer therapies. With the current pioneer researches on the tumor-suppressed function, structural basis, and regulatory mechanism of LACTB, the perspective hints at a great appeal of enzymic property, polymerization, mutation, and epigenetic and post-translational modifications in investigating LACTB’s role in cancer pathogenesis. This perspective provides novel insights for LACTB as a metabolic regulator with potential to develop targeted cancer therapies or neoadjuvant therapeutic interventions.

## 1. Introduction

**Figure 1:**
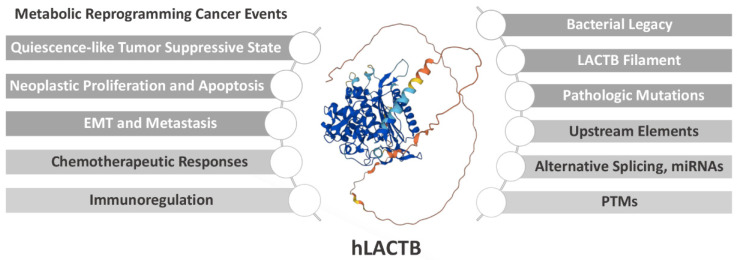
The frame of the perspective. The roles of LACTB in metabolic reprogramming cancer evets have been reviewed in the perspective. Moreover, the structural basis for its enzymatic function as well as the upstream regulation at transcriptional, post-transcriptional, and post-translational levels are elucidated further. The image of hLACTB is obtained by AlphaFold Protein Structure Database [6]. hLACTB, human LACTB; PTMs, post-translational modifications.

The shared biochemical features between mitochondria and Gram-negative bacteria, including DNA organization, core metabolism, and double-membrane architecture, are elucidated by the endosymbiont hypothesis [1]. Serine beta-lactamase-like protein (LACTB) is the exclusive mammalian mitochondrial homolog evolved from penicillin-binding proteins and β-lactamases (PBP-βLs) in bacteria [2]. The role of LACTB, the bacterial legacy, has consequently been switched from synthesizing the peptidoglycan layer to mediating lipid metabolism and tumorigenicity in mammalians [2,3,4]. This perspective provides novel insights on LACTB in cancer progression and clinical prognosis and developing targeted cancer therapies or neoadjuvant therapeutic interventions via elucidating LACTB’s influence on diverse cancers and involvement in the anticancer chemotherapeutic and immunotherapeutic responses; performing a molecular dissection of LACTB to streamline its role on reprogramming lipid metabolism, a hallmark in cancer [5]; and revealing relevant regulatory mechanisms (Figure 1).

## 2. Potential Prospects of LACTB in Cancer Applications

Rogue cells ready the body for tumorigenesis. The bona fide tumor suppressor gene makes a difference. In most conditions, LACTB is negatively associated with the growth of rogue cells while having a minimal effect on non-tumorigenic cells, and a low LACTB expression predicts poor prognosis. However, in nasopharyngeal carcinoma (NPC) and pancreatic adenocarcinoma (PAAD), LACTB is highly expressed and correlated with poor patient survival [7,8]. The relationship between the LACTB content and cancer development is reviewed in Table 1.

**Table 1:** The roles of LACTB in cancers and related mechanisms.

Cancer	Phenotype and Effect	Mechanism	Ref
Breast cancer	LACTB expression is downregulated in breast cancer and associated with malignancies; Knockdown of miR-374a suppresses the cell proliferative and colony-formation activity as well as migration and invasion capacity in vitro, and LACTB silencing reverses this change.	LACTB expression is downregulated by miR-374a, promoting cancer progression in breast cancer.	[9]
Breast cancer	LACTB expression is downregulated in breast cancer cell lines and tissues; LACTB drives the tumor suppressive mitochondrial state, reduced proliferation, and increased differentiation; Low LACTB, high mutant LACTB expression, or a molecular context with dysfunctional LACTB leads to enhanced proliferation and reduced differentiation.	LACTB inhibits phosphatidylserine decarboxylase and reduces the abundance of phosphatidylethanolamine and lyso-phosphatidylethanolamine, leading to a mitochondrial state compatible with tumor suppression, decreased proliferation, and enhanced differentiation.	[4]
Colorectal cancer	LACTB expression is downregulated in colorectal cancer; LACTB is associated with metastasis and advanced clinical stage; Low LACTB expression is associated with poor overall survival; Ectopic LACTB expression suppresses colorectal cancer cells proliferation, migration, invasion, and epithelial-to-mesenchymal transition in vitro, while knockout of LACTB results in an opposite phenotype; Ectopic LACTB expression inhibits colorectal cancer growth and metastasis in vivo, while knockout of LACTB results in an opposite phenotype.	LACTB directly binds to the C-terminus of p53 to inhibit p53 degradation by preventing E3 ubiquitin-protein ligase Mdm2 from interacting with p53; The antitumorigenic effects of LACTB overexpression is counterbalanced by p53 ablation in colorectal cancer; The downregulated expression of LACTB is due to promoter methylation and histone deacetylation.	[10]
Colorectal cancer	LACTB expression in colorectal cancer tissue samples is lower than that in nonmalignant tissue samples; LACTB inhibits cell invasion, migration, and proliferation by promoting autophagy in vitro; LACTB modulates tumorigenesis in vivo.	LACTB regulates the activity of phosphoinositide-3-kinase regulatory subunit 3, influences the level of phosphoinositide 3-kinases, and promotes autophagy via the protein kinase B/rapamycin signaling pathway.	[11]
Colorectal cancer	LACTB expression is lower in colorectal cancer tissue than that in the adjacent tissue; LACTB is associated with clinical stage in colorectal cancer; LACTB expression is lower in the colorectal cancer patients with lymph node metastasis than those without lymph node metastasis; Low LACTB expression is associated with a lower 5-year survival rate in colorectal cancer patients compared to those with high LACTB expression.	Not mentioned.	[12]
Colorectal cancer	Silencing LACTB promotes the viability, migration, and invasiveness in colorectal cancer cells; Silencing LACTB partially abolishes the apoptotic effect of pinocembrin on colorectal cancer cells.	Silencing LACTB enhances the expression of Matrix metalloproteinase-2 and N-cadherin but inhibits that of E-cadherin.	[13]
Colon cancer	LACTB mRNA expression is lower in colon cancer than in normal tissue; Overexpressing miR-1276 in colon cancer cells increases proliferation, migration, invasiveness, and epithelial-to-mesenchymal transition and decreases apoptosis, while supplementing LACTB suppresses these effects of miR-1276.	LACTB is inhibited miR-1276 inhibits in colon cancer cells, which inhibits autophagy.	[14]
Gastric cancer	LACTB is downregulated in the oxaliplatin-resistant MGC-803 cells; Overexpressing LACTB reduces the resistance of gastric cancer cells to oxaliplatin.	LACTB overexpression induces apoptosis via reducing the mitochondrial membrane potential and accelerating reactive oxygen species accumulation in the oxaliplatin-resistant MGC-803 cells; LACTB overexpression decreases glucose uptake and ATP synthesis, induces mitochondria and DNA damages, and inhibits autophagy of oxaliplatin-resistant MGC-803 cells.	[15]
Gastric cancer	No correlation is found between LACTB protein expression and clinicopathological indices of gastric cancer, including sex, age, histological differentiation, tumor location, Borrmman type, and TNM stage; LACTB expression is decreased in the gastric cancer tissues following oxaliplatin plus S-1 neoadjuvant chemotherapy; LACTB expression decreases in 68.6% patients, while LC3 increases in 60.8% following oxaliplatin plus S-1 neoadjuvant chemotherapy; High LC3 expression and low LACTB expression are associated with a poor response of patients with advanced gastric cancer to oxaliplatin plus S-1 neoadjuvant chemotherapy.	The expressions of LACTB and LC3 are negatively correlated following oxaliplatin plus S-1 neoadjuvant chemotherapy.	[16]
Glioma	LACTB expression is decreased in glioma; Low LACTB expression is correlated with a poor prognosis of glioma patients; Overexpressing LACTB suppresses the proliferation, invasion, and angiogenesis of glioma cells.	Overexpressing LACTB inhibits the expression of Proliferating cell nuclear antigen, Matrix metalloproteinase-2, Matrix metalloproteinase-9, and vascular endothelial growth factor.	[17]
Hepatocellular carcinoma	The mRNA and protein expressions of LACTB are down-regulated in hepatocellular carcinoma; Low LACTB expression is associated with TNM stage, histologic grade, and overall survival of patients; Overexpressing LACTB inhibits proliferation, invasion, and migration in vitro and decreases tumor growth in vivo.	LACTB is markedly correlated with genes involved in the lipid metabolism pathway indicated by online prediction, including fibrillin-1, carnitine O-palmitoyltransferase 1, medium-chain specific acyl-CoA dehydrogenase, phospholipid-transporting ATPase ABCA1, ferrochelatase, peroxisome assembly protein 12, E3 ubiquitin-protein ligase UBR1, and lathosterol oxidase.	[18]
Leukemia	Total LACTB is highly expressed in HL60 cells and low expressed in Raji cells; LACTB has multiple splicing isomers in leukemia cell lines, including XR1, V1, V2, and V3; The expression of V1 is high in U937 cells and low in Raji cells, that of V2 is high in HL60 cells and low in Raji cells, and V3 is poorly expressed in THP-1 cells.	Not mentioned.	[19]
Lung cancer	LACTB expression is downregulated in lung cancer tissues, while its methylation level is increased; Patients with high LACTB level exhibit improved survival; Overexpressing LACTB inhibits cell migration and invasion and induces apoptosis in H1299 and H1975 cells, while knocking down LACTB has opposite effects; LACTB negatively regulates the epithelial-to-mesenchymal transition process in lung cancer; LACTB strengthens the anticancer role of docetaxel in lung cancer; i.e., LACTB combined with docetaxel leads to a higher apoptotic rate and more potent inhibitory effects on H1299 and H1975 cells compared to docetaxel.	Knockdown of LACTB decreases the expression of E-cadherin (an epithelial cell-derived marker) and increases that of N-cadherin and vimentin (stromal cell-derived markers); Overexpressing LACTB increases the expression of E-cadherin and decreases that of N-cadherin and vimentin.	[20]
Melanoma	LACTB expression is low in melanoma tissues and cell lines; Low LACTB expression is correlated with short survival time in melanoma patients; Overexpressing LACTB suppresses the proliferation, migration, and invasion of melanoma cells in vitro; Overexpressing LACTB induces cell apoptosis and cell cycle arrest at the G2/M phase; Overexpressing LACTB suppresses the tumorigenicity and lung metastasis of MUM2B uveal melanoma cells in vivo.	Sex-determining region Y (SRY)-related HMG box-containing factor 10 binds to the promoter of LACTB and negatively regulates its transcription; Overexpressing LACTB prevents Yes-associated protein from translocating to the nucleus; LACTB directly binds to protein phosphatase-1, attenuates the interaction between protein phosphatase-1 and Yes-associated protein, and decreases the consequent dephosphorylation of Yes-associated protein.	[21]
Melanoma	LACTB expression is downregulated in melanoma tissues compared to the normal epithelium; Decreased LACTB expression predicts poor prognosis; iDPP/LACTB nanocomplexes promote cell apoptosis and block the cell cycle in vitro; iDPP/LACTB nanocomplexes inhibit tumor proliferation and induce cell apoptosis, consequently inhibiting melanoma growth in the subcutaneous B16-F10 melanoma model.	iDPP/LACTB nanocomplexes upregulates the p53 pathway and increases the mRNA expression of apoptosis- and cell cycle-related genes, including p21, BCL2-associated X, BH3-interacting domain death agonist, p53-induced death domain protein 1, and apoptosis regulatory protein Siva.	[22]
Nasopharyngeal carcinoma	Elevated LACTB expression is correlated with malignant behaviors and poorer survival of with multiple types of malignancies in nasopharyngeal carcinoma; LACTB expression is upregulated in high-metastatic nasopharyngeal carcinoma cells with reduced methylation in the promoter region; Overexpressing LACTB in the nasopharyngeal carcinoma cells promotes motility in vitro and metastasis in vivo, and knock-down of LACTB reduces metastasis of nasopharyngeal carcinoma cells; LACTB does not influence the proliferation of nasopharyngeal carcinoma cells.	LACTB activates Erb-B2 receptor tyrosine kinase 3/Epidermal growth factor receptor-extracellular signal-regulated kinase signaling, promoting the metastasis of nasopharyngeal carcinoma; Suppressing LACTB reduced nasopharyngeal carcinoma cellular motility by enhancing histone H3 stability and its acetylation.	[8]
Pancreatic adenocarcinoma	The mRNA and protein expressions of LACTB are high in pancreatic adenocarcinoma; Elevated LACTB mRNA expression in pancreatic adenocarcinoma patients is associated with a poor survival rate.	High LACTB expression is correlated with cell cycle-related genes and multiple immune marker sets.	[7]

### 2.1. LACTB Drives a Quiescence-like Tumor Suppressive State

A rogue cell escapes a normal behavioral control and acquires progenitor properties, sharing its abnormal behaviors or capabilities. Metabolic reprogramming is the driving force for cancer transformation and drug resistance. LACTB alters the phospholipid composition via functionally connected with phosphatidylserine decarboxylase (PISD), which converts phosphatidylserine (PS) to phosphatidylethanolamine (PE) and regulates the quantity of their lyso-forms [4]. Lyso-phosphatidylethanolamine (Lyso-PE, LPE) modulates the differentiation of neuronal PC12 [23] and cardiac cells [24]. Silencing LACTB increases the quantity of PE/LPE in mitochondrial membranes, which facilitates cellular proliferation and upregulates CD44 (cancer stem cell maker), while restoring LACTB expression reduces the PISD activity as well as the accumulation of PE/LPE, leading to a quiescence-like tumor suppressive state [4,25].

### 2.2. LACTB Mediates Neoplastic Proliferation and Apoptosis

The rogue cells have a high replication rate, which requires a high metabolism and a great deal of subtracts for DNA double helixes and phospholipid bilayers. LACTB directly binds to the C-terminal of p53, preventing p53 from degradation and consequentially impacting DNA-break repair and promoting apoptosis in colorectal cancer (CRC) [10]. Parallelly, melanoma-targeted nanoparticle iDPP-delivered LACTB upregulates p53 signaling pathway, promotes cell apoptosis, and blocks the cell cycle [22]. p53 exerts control over glucose and amino acid metabolism as well as lipid droplet formation [26]. The reprogrammed lipid metabolism has been widely recognized as an emerging hallmark of malignant cells in addition to Warburg effect [5]. Moreover, LACTB has exhibited an inhibitory role in breast cancer (BRCA) cellular proliferation via regulating the accumulation of the PE/LPE lipid species [4].

### 2.3. LACTB Mediates Epithelial–Mesenchymal Transition and Metastasis

Epithelial–mesenchymal transition (EMT) is a key step in carcinogenesis and confers metastasis by enhancing mobility and invasion [27]. EMT is regulated by lipid metabolism, and intervening cholesterol metabolism overcomes EMT-associated drug resistance [28]. Moreover, knockdown of LACTB decreases the expression of the epithelial cell-derived markers and increases the expression of stromal cell-derived markers in lung cancer, while overexpressing LACTB inhibits the migration and invasion of H1299 and H1975 cells, suppressing carcinogenesis [20]. However, LACTB expression is elevated in the NPC tissue compared to the non-cancer nasopharyngeal tissue, which is correlated with malignant behaviors and poorer survival in NPC [8]. Overexpressing LACTB promotes metastasis, and knockdown of LACTB reduces the metastasis of NPC cells [8]. The inconsistency of the LACTB’s roles between lung cancer and NPC may be related to the enzymic properties of LACTB, involving the polymerization dynamics (discussed in Section 3.2) and post-translational modifications (PTMs, discussed in Section 4.3) of LACTB.

### 2.4. LACTB Predicts Chemotherapeutic Responses

Chemotherapy is a predominant anticancer therapy. However, some cancer patients develop resistance after an initial treatment [29]. Identifying genes involved in chemotherapeutic responses is crucial for predicting antitumor response and treating drug-resistant cancer patients. Death receptor 4 (DR4) is palmitoylated and translocated into lipid rafts in circulating tumor cells, which is associated with increased oxaliplatin resistance and elevated efficacy of TRAIL liposomes [30]. LACTB has been associated with oxaliplatin-resistance [15] and oxaliplatin plus S-1 neoadjuvant chemotherapeutic response [16]. Moreover, it regulates the efficiency of pinocembrin [13] and strengthens the effect of docetaxel [20]. For instance, LACTB inhibits cell migration and invasion and suppresses EMT under the administration of docetaxel in lung cancer patients [20]. Hence, targeting lipid metabolism by combing traditional chemotherapeutic drugs is a promising strategy to overcome drug resistance in cancer, and LACTB is an effective lipid metabolic switch in chemotherapeutic responses.

### 2.5. LACTB Is Potentially Involved in Immunoregulation in Cancer

**Figure 2:**
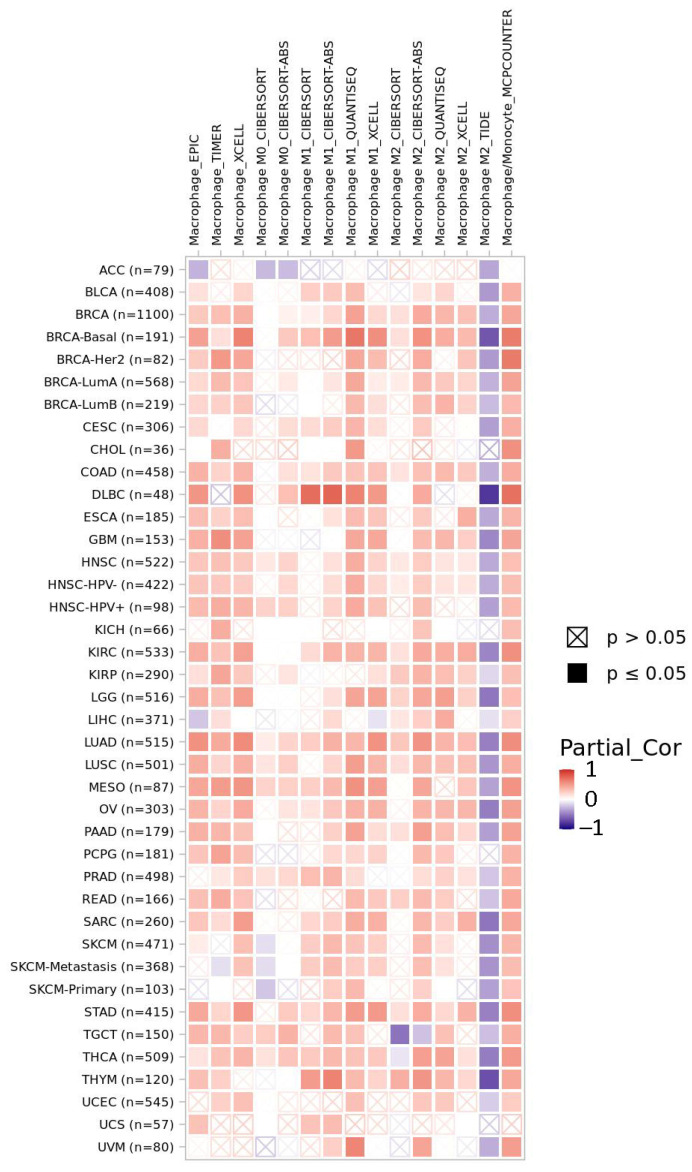
The correlations of LACTB expression with macrophage infiltration level are visualized in diverse cancer types by TIMER2.0. The heatmap shows the purity-adjusted spearman’s rho across various cancer types. Abbreviations: ACC, adenoid cystic carcinoma; BLCA, bladder urothelial carcinoma; BRCA, breast invasive carcinoma; CESC, cervical squamous cell carcinoma and endocervical adenocarcinoma; CHOL, cholangiocarcinoma; COAD, colon adenocarcinoma; DLBC, lymphoid neoplasm diffuse large B-cell lymphoma; ESCA, esophageal carcinoma; GBM, glioblastoma multiforme; HNSC, head and neck squamous cell carcinoma; KICH, kidney chromophobe; KIRC, kidney renal clear-cell carcinoma; KIRP, kidney renal papillary cell carcinoma; LGG, brain lower-grade glioma; LIHC, liver hepatocellular carcinoma; LUAD, lung adenocarcinoma; LUSC, lung squamous cell carcinoma; MESO, mesothelioma; OV, ovarian serous cystadenocarcinoma; PAAD, pancreatic adenocarcinoma; PCPG, pheochromocytoma and paraganglioma; PRAD, prostate adenocarcinoma; READ, rectum adenocarcinoma; SARC, sarcoma; SKCM, skin cutaneous melanoma; STAD, stomach adenocarcinoma; TGCT, testicular germ cell tumors; THCA, thyroid carcinoma; THYM, thymoma; UCEC, uterine corpus endometrial carcinoma; UCS, uterine carcinosarcoma; UVM, uveal melanoma.

Immunotherapy enhances the body’s immune response and inhibits immune escape [31]. Targeting macrophages holds great promise for cancer immunotherapy since macrophages account for 30% to 50% of the infiltrating immune cells in the tumor microenvironment (TME) compared to the low infiltration of T cells [32]. During apoptosis, PS is translated to the outer leaflet of plasma membrane, activating phagocytes and provoking apoptotic cell clearance, while their disruption results in secondary necrosis and immune activating danger signal release [33]. In the TME, PS is significantly increased, sharing innate immunosuppressive properties and facilitating tumorigenesis and progression [34]. The serine protease domain of LACTB has been proposed to participate in proteolytic signaling cascades, such as mitochondria-mediated apoptosis [3]; furthermore, LACTB participates in the PS metabolism [4], manifesting its involvement in immunoregulation in cancer. Moreover, in atherosclerotic plaques, LACTB expression is upregulated [35]; on the other hand, inhibiting LACTB attenuates the secretion of monocyte chemoattractant protein-1 (MCP-1) in lipopolysaccharide (LPS)-stimulated THP-1 macrophages [35], a crucial chemokine upregulated in inflammatory diseases [36]. Hence, LACTB may exhibit a pro-inflammatory effect in the macrophage-based anticancer therapy, and the correlations of LACTB expression with macrophage infiltration level are visualized in various cancers (Figure 2).

## 3. Structural Basis for the Enzymatic Properties of LACTB

The emerging roles of mammalian LACTB in metabolic signaling [37,38,39] prompt the explorations regarding the structural basis of its functionality, i.e., the enzymic properties. Cancer pathogenesis and progression have been relevant to the alterations in mitochondrial metabolism [40]. Recently, the cryo-electron microscopy (Cryo-EM) structure of LACTB filaments was reviewed by Cascone et al. [41]. Herein, this article focuses on the comprehensive impacts of LACTB on cancers and attends to undermine the structural basis for the catalytic activity of LACTB to elucidate its role in cancer events.

### 3.1. Mammalian Mitochondrial LACTB Shares Conserved Active-Site Motifs with Bacterial Class B Low-Molecular-Weight PBP-βLs

LACTB, a mammalian mitochondrial PBP-βLs homolog, contains three active-site motifs, i.e., -SXXK-, -[SY]X[NT]-, and -[KH][ST]G- [2]. LACTB is of a moderate sequence similarity (16–27%) with bacterial class B low-molecular-weight PBP-βLs (LPBP-B) [2], which is the first reported vertebrate example of microbial peptidase family [42]. In addition, the phylogenetic analysis indicates that the LACTB orthologs cluster with LPBP-B genes from free-living α-proteobacteria [42]. PBP-βLs function in synthesizing peptidoglycan, the major cell wall constituent in most bacteria [2]. β-lactamases governs penicillin metabolism, converting penicillin to penicilloate, while the LACTB single-nucleotide polymorphism (SNP) rs2729835 has been marginally associated with self-reported penicillin allergy in patients [43]. Hence, during eukaryote evolution, the prokaryotic PBP-βLs are conferred to the higher metazoan species and endowed with novel biochemical properties [2], i.e., the mammalian homolog LACTB in the mitochondrial intermembrane space (IMS) [44]. LACTB harbors a N-terminal region with a length of 50–100 amino acids and of no sequence similarity to the bacterial PBP-βLs, where a predicted mitochondrial import sequence starts [2], protecting LACTB from autoproteolysis [3]. In mitochondria, LACTB is polymerized into stable filaments with a length extending more than a hundred nanometers [44], promoting intramitochondrial membrane organization and micro-compartmentalization and therefore affecting mitochondrial metabolon organization.

### 3.2. Tertiary Structure of LACTB Underlies Its Enzymic Properties

The causative link between LACTB and obesity has been detected by data integration [45] and validated in the transgenic mice; i.e., LACTB overexpression results in obesity phenotype [46]. However, endurance exercise training downregulates LACTB in high-molecular-mass complexes (>600 kDa) without affecting the LACTB expression in the total proteome of skeletal muscle [47], which suggests the LACTB polymers (filaments) rather than total LACTB content within mitochondria may have conferred the metabolic advantages [48]. In this way, the human LACTB (hLACTB) filament formation may have played a critical role in cancer development and progression as well as anticancer therapeutic responses.

The tertiary structure of LACTB is intimately associated with its catalytic activity. It has been revealed by the high-resolution structure of a wild-type (WT) hLACTB filament, a middle region deletion mutant (in the absence of the catalytically important region), and an inhibitor peptide-bound filament [48]. Briefly, the middle region of the filament is required to perform the hydrolysis function, and hLACTB polymerizes via three distinct interfaces. The cleavage activity and polymerization dynamics of hLACTB are influenced by the pH value in IMS (ranged pH < 6–pH 7.4 in normal physiological conditions): the cleavage activity peaks at pH 8.6 and decreases along with pH, while the polymerization decreases at pH 6.0 compared to that at pH 7.4. Moreover, the alkaline pH, one of the unique mitochondrial characteristics in pathological cancers [49], may have facilitated the cleavage activity of LACTB. In this way, the increased/decreased total LACTB expression may not justify the altered behavior and enzymatic activity of LACTB in multi-cancerous events.

### 3.3. Pathologic Mutations of LACTB Dump Its Catalytic Activity

hLACTB polymers further assemble a double helix for substrate delivery and or product release, with amino acids surrounding S163, Y323, and H485, forming a positively charged plane and functioning as a catalytic center for peptide cleavage [48]. Moreover, the subtract selectivity of hLACTB was revealed by the hLACTB filament structure with an inhibitor peptide Z-AAD-CMK bound at 3.0-, 3.1-, and 2.8-Å resolution; i.e., the potential hydrogen bonds are formed between three residues in hLACTB (Y223, T325, and K394) and the aspartic acid residue in the side chain of Z-AAD-CMK [48]. H222 and G488 form potential hydrogen bonds with the backbone of Z-AAD-CMK, while F287 and L292 may have hydrophobic interactions with Z-AAD-CMK [48]. However, the LACTB mutations have been mapped in various cancers [48], e.g., E121K in BRCA, R371K in lung cancer, and E457K in urinary bladder cancer. These mutations may have a severe impact on the polymerization of hLACTB [48], which consequentially reduces its substrate affinity, dumps the V_max_ of its cleavage activity, and reprograms cancer lipid metabolism. Hence, mutation may be another angle in studying LACTB’s role in cancer.

## 4. Regulatory Mechanisms of LACTB

Given LACTB’s roles in cancer pathogenesis and therapeutic responses, understanding about its regulatory mechanisms is critical for developing precise and effective LACTB-targeted cancer therapeutics. More specifically, the upstream signaling, LACTB promotor activity, alternative splicing, microRNA (miRNA) regulation, and PTMs may play decisive roles in the expression and catalytic activity of LACTB in cancer tissues.

### 4.1. Transcriptional Events

Sex-determining region Y (SRY)-related HMG box-containing factor 10 (SOX10) participates in melanoma genesis [50]. Ten SOX10 binding sites have been predicted in the LACTB promoter [21]. It has been evidenced that SOX10 negatively regulates LACTB’s transcription in melanoma cells by binding to its promoter [21]. Moreover, LACTB responds to acute insulin signaling in skeletal muscle [38], which in turn emphasizes LACTB’s role in anabolism [51].

Along this line of consideration, the upstream elements make a difference. The LACTB mRNA expression has been positively correlated with histone H3 acetylation in the CRC cell lines, including FHC, ICECs, HCT116, HCT8, HT29, Caco2, SW480, SW620, and DLD1 [10]. Methylation and histone acetylation determine whether genes are on or off [52]. Promoter methylation and histone H3 hypoacetylation downregulate LACTB expression in CRC [10]. Moreover, the administration of 5-aza-2′-deoxycytidine (5-Aza-dC, a DNA-methyltransferase inhibitor) and trichostatin A (TSA, a histone deacetylase inhibitor) restore the LACTB expression in a dose-dependent manner, respectively [10].

### 4.2. Post-Transcriptional Regulators

Five hLACTB transcripts in the NCBI database suggest the relationship between alternative splicing and functional diversity of LACTB. When different mRNAs are assembled from a single gene, alternative splicing occurs by joining exons in different ways [53]. Splicing perturbation is common and associated with mutations in cancer [54]. Furthermore, a variety of splice isomers of LACTB have been found in the leukemia cell lines with diverse expression patterns [19].

As a noncoding RNA, miRNA acts as a crucial post-transcriptional regulator in gene expression [55]. A mature miRNA expression plays a causal role in tumorigenesis and progression; e.g., upregulating oncogenic miRNAs blocks tumor suppressor genes and leads to tumor formation [55]. LACTB has been regulated by multiple miRNAs, including miR-125b-5p [35], miR-351-5p [51], miR-1276 [14], and miR-374a [9]. The miR-125b-5p regulates inflammatory responses and atherosclerosis by targeting LACTB 3′UTR, which directly inhibits LACTB protein and mRNA expression and thereby reduces the expression of monocyte chemotaxis protein-1 (MCP-1) [35]. Moreover, miR-351-5P is involved in the transformation of muscle fiber types, i.e., promoting the proliferation of C2C12 myoblasts and inhibiting the formation of slow contraction fibers, via targeting LACTB 3′UTR [51]. In BRCA, miR-374a downregulates the protein expression of LACTB and promotes tumor metastasis [9]. Parallelly, in colon cancer, miRNA-1276 promotes cell proliferation by inhibiting LACTB [14].

### 4.3. Post-Translational Modifications

Protein PTMs, such as phosphorylation, methylation, and acetylation, affect almost all aspects of normal cell biology and pathogenesis [56]. A variety of PTMs are known to be altered in cancer development, which are potentially significant biomarkers in cancer diagnosis and prognosis [56]. LACTB has been subjected to post-translational regulation. Under general circumstances, the serine residues in LACTB are phosphorylated, which hints at LACTB’s response to specific phosphoprotein phosphatases [39]. During starvation, lysine acetylation of LACTB has been detected in the liver, which suggests that LACTB is regulated by a highly conserved acetyltransferase/deacetyltransferase pathway, resembling other key enzymes in metabolism [57]. In addition, in lung cancer, the methylation level of LACTB is elevated though its total expression is decreased [20], which hints at the importance of PTMs of LACTB and its promotor in cancer research.

## 5. Summary

The mitochondrial protease LACTB regulates lipid metabolism, specifically decreasing the accumulation of PE/LPE lipid species. The effects of LACTB in cellular proliferation and EMT have been reviewed in the perspective as well as its influence in the chemotherapeutic responses and immunoregulation. In most cases, LACTB expression is negatively associated with cancer progression, but inconsistency exits. The structural basis of LACTB underlies its enzymic properties and the upstream signaling exhibits transcriptional, post-transcriptional, and post-translational regulations on the expression and activity of LACTB, which hint at a great appeal of catalytic activity and function, polymerization dynamics, mutation, and polymorphism as well as epigenetics and PTMs in studying LACTB’s role in cancer pathogenesis rather than its simply total expression. This perspective provides novel insights on the LACTB’s metabolic role in cancer progression and clinical prognosis for developing LACTB-targeted cancer therapies or neoadjuvant therapeutic interventions, e.g., to combine the LACTB-targeted strategy with the developing drug delivery systems, such as the transferrin/α-tocopherol modified poly(amidoamine) dendrimers to improve tumor targeting [58] or transferrin and octaarginine modified dual-functional liposomes to enhance intracellular delivery [59].
